# Some Observations from the Life of the Late Dr. John M. Riggs, of Hartford, Conn.

**Published:** 1887-03

**Authors:** Geo. A. Mills

**Affiliations:** New York


### SOME OBSERVATIONS FROM THE LIFE OF THE LATE DR. JOHN M. RIGGS, OF HARTFORD, CONN.

BY GEO. A. MILLS, NEW YORK.

ABSTRACT OF PAPER READ BEFORE THE CONNECTICUT VALLEY DENTAL SOCIETY, AT THEIR ANNUAL MEETING, HELD IN HOLYOKE, MASS., OCTOBER 14TH AND 15TH.*

My first meeting with Dr. Riggs was at a gathering of dentists in the city of Hartford, in the year 1865. Up to this date, if I am rightly impressed, he had mingled but little, if any, in the associated bodies of dentists. He was brought before the meeting at this time at the personal request of Dr. McManus, of Hartford, and in his introduction of Dr. Riggs, he referred particularly to the great interest the doctor had given to the disordered condition, so common in the mouth, and which had been generally regarded as a senile one, and not amenable to remedial agencies. It was also so regarded in all the medical and dental literature at that time. Dr. Riggs cordially accepted the invitation to meet with the Association and to participate in its proceedings, and addressed the society at some length, taking occasion to introduce the subject so much in his thought and practice at that time. As a mark of esteem he was elected President of the Association for that year, and from that time to the end of his earthly career he was an active or honorary member of a number of dental societies.

It must not be overlooked that before this date he had made an indelible impression for good in the city of Hartford, and had won a reputation as a practitioner far above the average of his fellows.

Dr. Riggs was of a sturdy, yeomanry type. His father was a farmer, and in early life he was inured to habits of industry. As a lad he learned the art of a stonecutter, and in this he excelled. A desire for mental culture took him to college, and by his trade, together with vacations spent in teaching, he supported himself while attending Trinity College, from which he graduated in 1837.

* This abstract was received from the Secretary of the society, Dr. Geo. A. Maxfield, some time since, but it was impossible to make space for it until now.—EDITOR.

Dentistry, in its poverty at that day, needed the pioneer spirit and enthusiasm of such an one as John M. Riggs, and he was led directly to become one of its abettors by the counsel of Dr. Horace Wells, who was then practicing dentistry, and who has since been honored, first by our mother profession of medicine and surgery, and later, by the citizens of his own State, in the bronze statue which stands in the city park of Hartford.

We must not deny to Dr. Riggs his share in the honor bestowed upon Dr. Wells, both in the part he took in the discovery of anæsthesia, and in the co-operative work of the original designing of the classical face of the statue that records his honor. With the first incident many dentists are familiar. It consisted in the extracting of a tooth from the mouth of Dr. Wells by Dr. Riggs, while he was under the effect of the anæsthetic administered by himself. It was this circumstance on which was based the claim of the discovery of anæsthesia, and which stands to-day as a recorded historic fact. The second incident is not so well known. Several days after the death of Dr. Wells, while his body was lying in the receiving tomb in Hartford, it occurred to Dr. Riggs that possibly the time would come when this discovery would be honored by the profession in some fitting memorial. He therefore requested permission of the widow to go to the tomb and take a mask mould of the doctor's face, and this he laid away as a hidden treasure, to serve its purpose as the future might decide. Dr. Riggs had, even before his commencement of professional life, evinced no small degree of skill as a sculptor, showing the possibilities of an artist had he made choice of such a calling, and how well this talent served him, the face now moulded in bronze will testify, as it was designed from this original cast.

The prime characteristic of the doctor's inner nature was integrity. By this I mean the sacredly following of what to him seemed to be rational and true, and capable of something approximating to a demonstration. This was markedly manifest throughout his professional life. No one who has ever stood by him and witnessed his clinics could fail to give full testimony that "faithfulness was engraved on the tablets of his thought." No such opprobrious remark as "I guess that will do" was ever heard from him. He had a trait of character that ruled his daily actions. While he was a tenacious defender of what seemed to him as truth, yet he counseled with expediency.

He was truly a type of the old-school practice, and he tenaciously held to it throughout his professional life. His practice in general might justly be termed radically conservative, as opposed to the radical idealist. To the dental student of the present day, the methods of preparing a cavity and placing gold within it that were the common practice in Dr. Riggs' day, would seem crude indeed. The principle of condensing gold, and the form of the instrument used, would prove, on close examination, to be the same as those now coming largely into favor, viz., lateral and rotary pressure.

He was an early and late advocate of what is termed by some "oral gardening." The sixth-year molar was watched with the scrutiny of an eagle's vision, and woe be to its existence in the dental arch if it intimated the least maldirection or impingement upon any of its fellows, thus occupying space which could, in his judgment, be more wisely appropriated by the others.

With a few casual exceptions here and there, Dr. Riggs did not seem to attract more than a momentary notice from our profession in general. To this Society belongs the credit of giving a stimulus to his purpose. This consisted in the commencement of clinical teachings. The first subject was Dr. Goodrich, at the regular meeting of this society, held in the city of Northampton, on the eleventh of June, 1867. At this time he was under the necessity of forming the instruments for the case in hand, and at this clinic a decided enthusiasm was manifested. Dr. Riggs has often alluded to this occasion in my presence, contrasting in the later years the change of expression regarding his theory and practice. This clinic was given publicity in quite lengthy detail through the columns of the *Springfield Republican*.

Two years later (June 10, 1869) this society passed a resolution giving credit to the doctor for originality in defining this disease. As the result of this introduction, it may be said that his *début* was made at this time. In 1870 he accepted an invitation from the Brooklyn Dental Society to give a series of clinics at the Brooklyn Dental Infirmary, which was at that time a protégé of that society. Not far from this date he gave some clinics before the Harvard Dental School. During this time, Mr. Charles Tomes visited this country, and at the Harvard school he met Dr. Riggs and saw his operations, and on his return home wrote an article upon the subject, confirming Dr. Riggs' views, giving it as his opinion that the etiology of the disease, regarded at that time as a premature wasting of the alveolar edge, would or might prove to have a likeness to caries, an opinion which the knowledge of the present day seems to conclusively confirm.

In 1875 Doctor Riggs was elected a member of a society in New York City, before which he read a paper embodying his views on the pathology and treatment of this disorder. This paper was published in the *Pennsylvania Journal of Dental Science.* As far as I know, this was the only article ever published over his signature distinctly embodying his views on this subject, although at different times he, in part, reiterated them. It was not till 1877, at a meeting of the American Dental Association, held in Chicago, that this subject was at all noticed by this body. The ball was set in motion by Dr. Rehwinkel, of Chillicothe, Ohio, under the title of "Pyorrhœa Alveolaris." There was an extended discussion of this subject on that occasion, and from that day to the present time the subject has not suffered for an airing. During all these years Dr. Riggs became more and more in demand for his special treatment, patients coming many hundreds of miles for his services. He had opportunity for the interchange of his views with individual dentists by correspondence and visitations, yet it was a source of no little disappointment to him that those in authority in the several dental schools did not take sufficient interest in it to have intelligent systematic teachings on this subject, but he predicted that the time would come when they could not resist the demand for such teaching. This prediction has proved true. One school, and only one, has provided for such teachings the coming season. It must be admitted that violent and sarcastic opposition to good and valuable things is nothing out of the usual course of human experience, for we know that those teachings which most concern the welfare of mankind have been most wickedly opposed, doubtless more through misconception than otherwise.

We cannot marvel that this boon of amelioration has met the same experience, but out of it there has come a greater acceptance of its value, and the future atmosphere that will be generated by the higher state of living will remove this gift far from selfishness, and the name of Dr. Riggs, as a benefactor, will shine out with clearness in the pages of historic records.

I do not present our brother as a perfect model, but only one stone in the hands of the builder, which, when it is fitly joined with others, will have its part in the grand and holy temple of which it was destined to form a portion.

Whatever evolution may do for the better understanding of the disorder brought to our notice by Dr. Riggs, nothing will or can disassociate his name from it. It stands as his bequest, and the faithful and unerring pen of history will so record it. If we are as faithful to the trust committed to us as he has proved to his, we will, in a large degree, become helpful servants to mankind.

